# Is It Possible to Earn Abnormal Return in an Inefficient Market? An Approach Based on Machine Learning in Stock Trading

**DOI:** 10.1155/2021/2917577

**Published:** 2021-12-08

**Authors:** Bui Thanh Khoa, Tran Trong Huynh

**Affiliations:** ^1^Finance, Economics, and Management Research Group, Ho Chi Minh City Open University, Ho Chi Minh City, Vietnam; ^2^FPT University, Hanoi, Vietnam

## Abstract

Risk management and stock investment decision-making is an essential topic for investors and fund managers, especially in the context of the COVID-19 pandemic. The problem becomes easier if the market is efficient, where stock prices fully reflect potential risk. Nevertheless, if the market is not efficient, investors may have an opportunity to find an effective investment method. Vietnam is one of the emerging markets; the efficiency is still weak. Thus, there will be an opportunity for astute investors. This study aims to test the weak-form efficient market and provide a modern approach to investors' decision-making. To achieve that aim, this study uses historical data of stocks in the VN-Index and VN30 portfolio to buy and sell within a one-day period under the rolling window approach to test the Ho Chi Minh City Stock Exchange (HoSE) through a runs test and to perform stock trading using the support vector machine (SVM) and logistic regression. The buying/selling of stocks is guided by the forecasted outcomes (increase/decrease) of logistic regression and SVM. This study adjusted the return rate in proportion to the risks and compared it with index investments of VN-Index and VN30 to evaluate investment efficiency. The test results dismissed the weak-form efficient-market hypothesis, which opens up many opportunities for short-term traders. This study's primary contribution is to provide a stock trading strategy for short-term investors to maximize trading profits. Because logistic regression and SVM have proven effective trading methods, investors can use them to achieve abnormal returns.

## 1. Introduction

Risk management and stock investing decision-making are critical topics for investors and fund managers, particularly regarding the COVID-19 pandemic. If the market is efficient, where stock prices adequately represent a possible risk, the issue becomes simpler to solve [1, 2]. Some investors often use technical analysis to select stocks as historical data (mainly price and trading volume) in the short term. Some technical analysis tools forecast price movement direction, deciding whether to buy or sell stocks [3]. Mizrach and Weerts [4] used technical indicators, price, and volume history to forecast future stock returns, sometimes called “chartists” because they use graphical trading representations. Azzopardi [5] applied principles to study how human emotions impact financial decision-making. SVM and artificial neural networks (ANN) identify market abnormalities in many financial markets worldwide [6]. Nevertheless, Fama [7] proposed efficient-market hypothesis casts doubt on the reliability of the technical analysis. This theory will not help beat the market because it assumes that the price of a security fully reflects all available information [8–10]. That said, each market is efficient to a certain extent; specifically, there are three types of efficient markets in ascending order: weak, semistrong, and strong. Even in the weak form, the stock's price fully reflects its historical data.

For that reason, the security price cannot be predicted solely based on past prices [11]. Some empirical evidence suggests that markets are not truly efficient, which implies that investors may use templates or prediction models to achieve a higher rate of return [12, 13]. Hawaldar et al. [14] tested the weak-form efficient-market hypothesis of the Bahrain Bourse stock market for the period 2011 to 2015 and concluded that the Kolmogorov–Smirnov goodness-of-fit test, run test, and autocorrelation test reject the weak-form efficient-market hypothesis. Kumar et al. [15] supported India's weak-form efficient-market hypothesis for 2012–2017 but rejected the medium-form efficient-market hypothesis. Mensi et al. [16] studied the daily closing prices on the global and regional GIPSI stock markets in the USA and five GIPSI stock markets in Europe from January 1, 2009, to September 8, 2017. GIPSI, worldwide, and US markets are all inefficient, particularly in the short term. Whatever the time range, the Greek stock market is the most inefficient of all markets. In the short and long run, Portugal and Ireland have the least inefficient marketplaces. These findings also suggest that stock markets may not be suitable for risk diversification in asset allocation or risk hedging. The author also suggests that these findings have significant consequences for investors and policymakers. In reality, investors may utilize knowledge about long-term memory and the differential threshold for persistence across time horizons to outperform the market and generate abnormal returns.

A recent trend in behavioral finance theory is to explain that anomalies complement the shortcomings of the efficient-market hypothesis. Kahneman and Tversky, a pioneering researcher, point out that investors rely heavily on emotions and instincts rather than rationality to make decisions [17]. Emotional decision-making can lead to mistakes when making irrational investment choices. Some anomalies associated with behavioral finance theory include calendar, fundamental, and technical anomalies [18]. Some experiments show the weekend effect, holiday effect, turn-of-the-month effect, and January effect [19]. Rossi studied calendar anomalies in the Milan Stock Exchange from January 2005 to December 2015. They found that returns were negative on Monday and positive on Wednesday. Thus, investors should buy on Monday and sell on Wednesday. One limitation of these studies is that the effects may disappear or even reverse [20]. As a result, investors may be exposed to risks when using these investment trends.

This study aims to test the weak-form efficiency of the HoSE market and determine whether investors using logistic regression and the SVM model can outperform the market. The runs test approach rejects the weak form of an efficient market. These findings suggest that classic econometric and statistical models are likely to beat the market. However, the constantly evolving machine learning algorithms provide a viable alternative to traditional regression models. Some studies on the SVM application in finance have obtained many positive results, such as Cao and Tay [21], Huang et al. [22], Lu et al. [23], Mohamed [24], Azimi-Pour et al. [25], and Syriopoulos et al. [26]. The rolling window drives the buying and selling of securities by the logistic regression model's output and the SVM algorithm. Input variables include close (closing price); HL (the highest minus lowest price); LO (lowest price minus opening price); variation (the difference in closing price between 2 consecutive trading sessions); ma7, ma14, and ma21 (average price of 7, 14, and 21 consecutive sessions, respectively); sd7 (standard deviation of 7 consecutive sessions); vnc (the difference in closing prices of VN-Index for 2 consecutive sessions); vnipc (return rate of VN-Index portfolio); and insect (time trend). The data covers all stocks in the VN30 basket from January 28, 2000, to July 30, 2021. As a result, the SVM investment strategy beat the market with an extremely high average return rate.

Machine learning may discover weak-form efficient markets and develop trading methods for short-term investors, thereby maximizing earnings. Predicting the movement of stock prices using algorithms, such as the SVM model, has demonstrated a high accuracy. The parameters of the machine learning model were accurately predicted using the rolling window technique. Since a sample's representativeness may be impaired by a period too short or too lengthy, 365 days is a good choice for a historical data set. Stock investing in a weak market is usually tricky for short-term investors. The SVM model, in particular, is a valuable tool for predicting the direction of price movement in the market. It is necessary to modify the investment returns to reflect the inherent risks to raise the degree of trust in the investment performance review. The Sharpe ratio is used to manage risk, while the *T*-test is used to evaluate trading methods. Due to the SVM model's superb accuracy, the trading strategy employing it has produced a great return.

The following diagram depicts the flow of this study. Next, a brief review of relevant literature is provided: the efficient-market hypothesis (EMH), logistic regression, support vector machine (SVM).  Section 3 of the study provides the conceptual foundation for the paper, including the theories of weak-form efficient-market hypothesis testing and price movement forecasting decision.  Section 4 focuses on empirical data and outcomes.  Section 5 provides further in-depth explanations of the study's findings. In Section 6, the conclusions of this study and the limits and potential for further research are summarized and explained.

## 2. Literature Review

### 2.1. Efficient-Market Hypothesis (EMH)

Fama [11] first proposed EMH in the 1970s. This article is significant because it paved the way for many other studies on the accuracy of the EMH theory. The concept of efficiency refers to the rapid absorption of information instead of the resources that produce maximum output as in other fields of economics. Information is defined as news that can affect prices and is unpredictable. In capital markets, efficient markets can be interpreted in various ways. The market in which prices always reflect available information is called an efficient market [11]. Meanwhile, Malkiel [27] argued that a capital market is efficient if it wholly and correctly reflects all relevant information in determining security prices. Generally, however, markets are considered efficient for certain types of information if disclosing that information to participants does not affect stock prices. EMH includes the following hypotheses:

Weak-form efficiency hypothesis: this degree of efficiency exists when a security's price reflects historical data about a security's price, including stock price and trading volume. In other words, one can forecast current stock prices on past stock prices. Testing the weak-form efficient-market hypothesis mainly concerns whether there is a statistical dependence between price changes. In other words, if the price changes are random, the market is a weak-form efficient market. Several frequently used testing techniques are autocorrelation and Ljung–Box's *Q* [28], variance ratio, LM-test [29], CD-test [30], Wright's test [31], runs test, January effect, and unit root test [32].

Semistrong-form efficiency hypothesis: this degree of efficiency exists when a security's price reflects publicly accessible market information, including historical data on security prices and publicly available information in the market, such as those in an issuer's prospectus. The semistrong-form efficient market encompasses the weak-form hypothesis because all market information, including stock prices, interest rates, and trading volume, must be publicly analyzed using the weak-form efficient-market hypothesis. Public information includes all nonmarket data, such as earnings and dividend announcements, P/E ratio, D/P ratio, P/B ratio, stock splits, and political economy information. Studies examining semistrong-form EMH can be classified into these two categories:

- Studies that sought to forecast future rates of return using publicly accessible data, except for pure market data such as price levels and trading volumes, have been included in the weak-form test. These studies may include time series analysis of returns or cross-sectional distribution of returns of individual stocks. EMH proponents argue that it is impossible to use publicly available information to predict future returns using past returns or to forecast future cross-sectional distributions of returns (e.g., highest quartiles or deciles of returns) [33–36].
- Event studies investigate how quickly stock prices change in response to specific key economic events. One practical approach is to test whether it is feasible to invest in stocks and earn an extraordinarily high rate of return after a significant event (such as stock merges, stock splits, central economic data, and principal) is publicly announced or not. Again, EMH proponents expect stock prices to adjust rapidly so that investors cannot earn high returns by buying after public announcements and paying regular transaction costs [37–40].

Strong-form efficiency hypothesis: this degree of efficiency exists as all information is fully reflected in stock prices, including nonpublic information such as internal information. The strong-form efficient-market hypothesis combines both the weak-form and the semistrong-form efficient hypothesis. The strong-form efficient-market hypothesis extends the assumption of efficient markets, in which prices reflect publicly available information to a perfect market, and all information is free and available. It is necessary to know when internal or insider information arises to evaluate strong-form efficient markets. This timing is hard to identify. Strong-form efficient markets are often researched in developed countries. For emerging markets, most studies focus on weak- and semistrong-form EMH. The exploration of strong form effectiveness is still a controversial matter among scholars [41–43].

### 2.2. Logistic Regression

Logistic regression is a statistical technique that describes the relationship between independent variables and binary dependent variables (which can also be applied to discrete dependent variables). Through this relationship, logistic regression allows the output prediction of a given set of input values. In predicting the output using logistic regression, this study calculates the probability that the output takes the value 1 with the given observation data to find *P*(*Y*=1*|X*). With the assumption of binomial distribution of the dependent variable, this study considers the odd ratio as follows:

(1) $$\begin{array}{c} G\left(X\right)=\frac{P\left(Y=1\;|\;X\right)}{P\left(Y=0\;|\;X\right)}=\frac{P\left(Y=1\;|\;X\right)}{1-P\left(Y=1\;|\;X\right)}. \end{array}$$

Taking the logarithm on both sides of (1), this study has

(2) $$\begin{array}{c} \ln\;G\left(X\right)=\ln\left(\frac{P\left(Y=1\;|\;X\right)}{1-P\left(Y=1\;|\;X\right)}\right)=X\beta , \end{array}$$

where *β*=(*β*_0_, *β*_1_, ..., *β*_*k*_)  that are the parameters to be estimated.

From equation (2), this study makes the equivalent transformation as follows:

(3) $$\begin{array}{c} P\left(Y=1\;|\;X\right)=\frac{e^{X\beta }}{1+e^{X\beta }}. \end{array}$$

Usually, the maximum likelihood estimation (MLE) method is used to estimate the parameter *β*. The classification rule is determined by equation (3) as follows: $y_{i}=\left\{\begin{array}{c} 1,P\left(y_{i}=1\;|\;X\right)\geq 0.5,\\ 0,P\left(y_{i}=1\;|\;X\right)<0.5. \end{array}\right)$

Logistic regression is applied in many fields for the binary dependent variable. In finance, Han et al. [44] used a sample of 76 firms and 32 variables related to their financial ratios to predict precarious financial situations. The authors used the backward stepwise method in logistic regression and obtained results with high accuracy of 92.86%. Konglai and Jingjing [45] used logistic regression to analyze listed companies' credit risk in China. The data used included 130 companies with 6 dependent variables and was divided into 90 companies for the training set and 40 for the testing set. The training sample has an accuracy of 87.8%, while the testing set has a precision of 75%.  Table 1 summarizes some publications that have used typical logistic regression.

### 2.3. Support Vector Machine (SVM)

The SVM algorithm was proposed by Vapnik and Lerner [50] to solve the classification issue. SVM is a supervised mathematical algorithm used to classify data in different dimensions. Suppose that *Y* is a categorical variable with two possible values –1 and 1 and *X* is an input variable. The classification hyperplane is defined by the equation: *wx*^*T*^+*b*=0, where *w* and *b* are the coefficients. The coefficients *w* and *b* should be chosen such that  *wx*^*T*^+*b* ≥ 1 if *y*_*i*_=1 and w*x*^*T*^+*b* ≤ −1 if *y*_*i*_=−1. The training set is used to find *w* and *b* such that ‖w‖ is minimized, and the vectors *x*_*i*_ in which |*y*_*i*_|(*wx*_*i*_^*T*^+*b*)=1 are called support vectors. To improve classifier efficiency, a kernel function is used to map the data to a high-dimensional space where the data will be more clearly segregated. The kernel function is defined by the dot product: *K*(*x*, *y*)=〈*f*(*x*), *f*(*y*)〉. Some common kernel functions are linear, polynomial, and radial basic function. Nevertheless, for some complex data sets, it is impossible to find a perfect hyperplane. Hence, Cortes and Vapnik [51] propose to add soft margins, that is, accepting some misclassified observations. The SVM algorithm is now minimized: min_*w*,*b*,*ξ*_(1/2*w*^*T*^*w*+*C*∑_*i*=1_^*n*^*ξ*_*i*_)  given that *y*_*i*_(*w*^*T*^*wϕ*(*x*_*i*_)+*b*) ≥ 1 − *ξ*_*i*_, where *C* is a hyperparameter and *ϕ* is a conversion mapping from low- to high-dimensional space.

SVM is often used in financial research. For instance, Kim [52] has used SVM to predict hotels' bankruptcy in Korea. Between 1995 and 2002, a sample of 33 hotels was collected, and the forecast results achieved 95% accuracy. In the Japanese market, Huang et al. [22] used SVM to predict the direction of the NIKKEI 225 Index and showed that SVM outperformed other classification methods in their study, including random walk model, quadratic discriminant analysis (QDA), and ANN. Ren et al. [45] integrated SVM with investor behavior analysis in the Chinese market. This study forecasted the SSE 50 Index's movement from 2014 to 2016 in 485 trading days, used both fivefold and rolling window methods, and reached a maximum accuracy of 89.93%.

## 3. Research Data and Methods

### 3.1. Research Data and Variable Description

Research data includes 30 companies in the VN30 basket (unadjusted price), VN-Index, and VN30 index in a one-day period.  Table 2 describes the tickers and their observations in the VN30 basket.

The data collection period was from July 28, 2000, to July 30, 2021, in which some companies were newly established, and there were some days off. Hence, the number of observations of these companies was varied. The data was collected from the website https://www.hsx.vn (Ho Chi Minh City Stock Exchange). Each observation included date, ticker, closing price, opening price, highest price, lowest price, and trading volume. The variables in the study are described in Table 3.

### 3.2. Research Method

#### 3.2.1. Testing the Weak-Form Efficient-Market Hypothesis

According to the weak-form efficient-market theory, a security's past prices cannot forecast current prices and generate abnormal returns. There are other testing techniques available, but these studies employ runs tests like some previous studies, including Fawson et al. [57], Moustafa [58], Ahmad et al. [59], Nisar and Hanif [60], Hamid et al. [61], and Wei [62]. The runs test, known as the Wald–Wolfowitz test, is a nonparametric statistical test that examines the randomness hypothesis on a two-state data series [63]. The runs test will assess whether the elements of the series appear independently. In other words, if assuming the price increases or stays the same as (+) and decrease as (–), then a weak-form efficient market implies that price changes are independent. When the sample size is large enough, the statistic $Z=R-\bar{R}/s_{R}∼N\left(0,1\right)$, where:

 
*R*: number of runs in the sample (each run is a sequence of consecutive “+” or “−” signs)
 
$\bar{R}$: expected value of *R*, calculated by the formula $\bar{R}=2n_{1}n_{2}/n_{1}+n_{2}+1$
 
*s*_*R*_^2^: the standard error of the runs, *s*_*R*_^2^=2*n*_1_*n*_2_(2*n*_1_*n*_2_ − *n*_1_ − *n*_2_)/(*n*_1_+*n*_2_)^2^(*n*_1_+*n*_2_ − 1) (*n*_1_, *n*_2_ are the number of “+” and “−” signs, respectively)

Mainly this method explores the randomness in the changes of the VN-Index and VN30 index. If this variation is random, it supports the weak-form efficient-market hypothesis, suggesting that traditional forecasting models using historical data are unlikely to produce an excess return.

Finally, to test the influence of factors affecting price movements, we performed logistic regression for all data in the research period. This result also implies that investors with little experience in academic knowledge can still base the fluctuations of variables (variables with strong impact) to make investment decisions.

#### 3.2.2. Price Movement Forecasting and Investment Decision-Making

**Figure 1:**
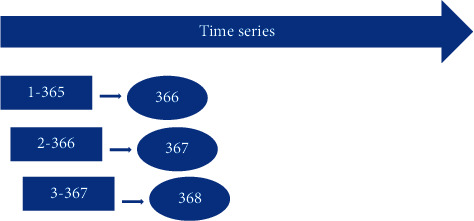
Rolling window method.

This study focuses on two models, logistic regression and SVM, to forecast price movement direction. Assuming that the historical data has a maximum value of 1 year, the study will use fixed training data of 365 observations to make forecasts using the “rolling window” method. Algorithms are used to identify the optimal parameters for the first 365 observations, forecast the 366th observation, and continue until the last observation, as shown in Figure 1.

### 3.3. Forecasting Model

(4) $$\begin{array}{c} \hat{{\text{foredir}}_{t+1}}=f\left({\text{close}}_{t}{\text{,HL}}_{t}{\text{,LO}}_{t}{\text{,variation}}_{t}{\text{,ma}7}_{t}{\text{,ma}14}_{t}{\text{,ma}21}_{t}{\text{,sd}7}_{t}{\text{,vnic}}_{\text{t}}{\text{,vnipc}}_{\text{t}}\text{,insec}\left(\right)\right). \end{array}$$

The sigmoid function is employed for the logistic regression model, and the MLE method is used to estimate the regression coefficients. For the SVM algorithm, the kernel function radial and *γ*=0.1 are used. Based on the logistic regression and SVM models, the investors will buy/sell stocks, respectively. To assess investment performance, this study adjusts risks using the Sharpe ratio [64, 65]: SharpeRatio=*r*_*p*_ − *r*_*f*_/*σ*_*p*_, where

 
*r*_*p*_: return rate of the portfolio (or security) *p*
 
*r*_*f*_: risk-free rate (1-year treasury note)
 
*σ*_*p*_: standard deviation of the portfolio (or security) *p*

Finally, this study compares the performances of investments made by the logistic regression model and SVM with investments made by the *T*-test according to VN30 and VN-Index. Furthermore, this study seeks to determine whether holding a single stock is more efficient than holding a market portfolio index. The novelty of this study is to provide a securities trading method using a logistic regression model and SVM.

## 4. Results

### 4.1. Descriptive Statistics

**Figure 2:**
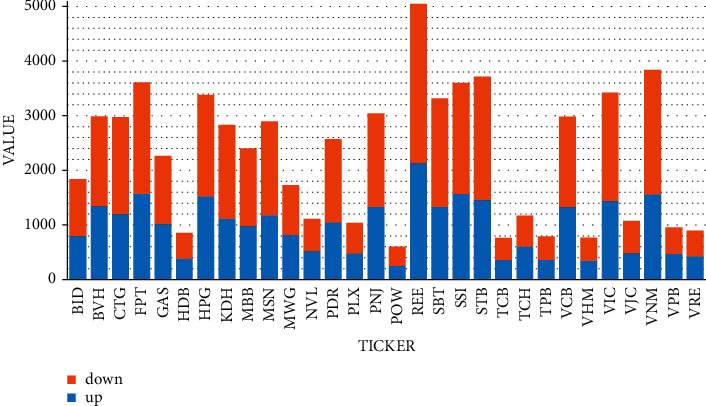
The number of observations on the tickers' price increases/decreases.

The descriptive statistics of the variables are described in Table 4 below. The table shows that the price fluctuates from 0.233 USD/share to 23.233 USD/share; the average price is 2.266 USD/share. The foredir ends up with 39,096 observations resulting in a decrease in closing price compared to the day before. The remaining 29,420 observations of closing price were not decreased; the specific amount is shown in Figure 2. The most muscular daily closing price movement-down 7.108 USD/share on a day, occurred to VNM ticker on July 5, 2007 (exchange rate USD/VND = 22,748).

**Figure 3:**
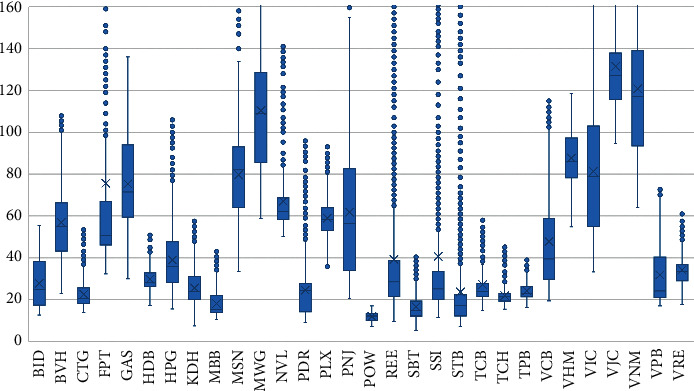
Closing prices movement of tickers.

**Figure 4:**
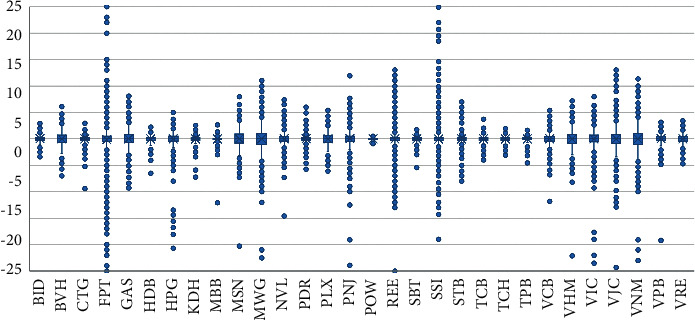
Variation movement of the tickers.

The fluctuations of the variables close and variation are better shown in the boxplot on Figures 3 and 4. Some tickers such as FPT, REE, SSI, and STB tilted to the right and had unusually high closing prices in some trading sessions. Still, the tickers' variation is mostly stable. This study noticed an anomaly that FPT plummeted 7.429 USD/share on May 21, 2007, the most profound fall across all stocks in the VN30 portfolio in all trading sessions. The decline in share price is due to FPT's dividend payment policy with a payout ratio of 2:1, which shows that one more FPT share will be awarded for every two FPT shares an investor holds.

**Figure 5:**
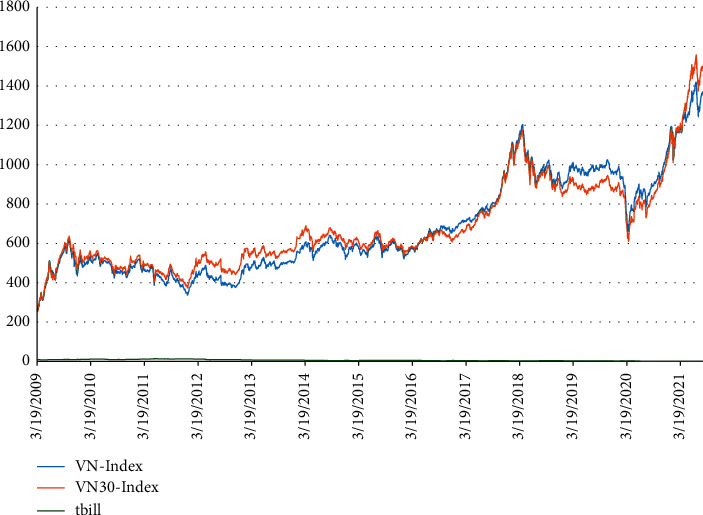
Movement of VN-Index, VN30 index, and bond interests (T-bill).

For the market, this study has a summary table detailing the variables closevn (closing price of VN-Index), vnic, vnipc, closevn30 (closing price of VN30 index), rvn30 (return rate of VN30 index), and rf (the interest of 1-year government bonds).  Table 5 and Figure 5 show that the closing prices of the VN-Index and VN30 index primarily fluctuate together, while bond interests are primarily stable and tend to decrease. From the beginning of 2020, this study noticed that both the VN-Index and VN30 dropped significantly and then rose again. This result was because of the COVID-19 pandemic, which obstructed the production and trading activities of businesses. When the businesses stabilized, the cash flow poured into the financial investments, leading to increased stock prices.

### 4.2. Runs Test Results

Runs test results showed that the weak-form efficient-market hypothesis is dismissed at 1%, implying that technical analysis can obtain an abnormal return.

### 4.3. Accuracy of Price Movement Forecasting Models

This study used the logistic regression model and SVM to forecast the increase and decrease of stocks based on the rolling window method. The accuracy value (the number of correct predictions out of the total predictions) is summarized in Table 6. The average accuracy in forecasting 30 stocks of the logistic regression model and SVM are 58.93% and 92.48%. The SVM model has proven to be more effective than the logistic regression model.

### 4.4. Stock Trading Results

Stocks were traded on the stock price increase and decrease forecasts made by the logistic regression and the SVM models. The results of average daily return before and after risk adjustment are in Table 7. As seen in Tables 5 and 7, the SVM model outperforms the logistic regression model and the portfolio index investment (including VN30 and VN-Index). To determine the efficacy of the trading strategies, this study conducted five one-sided *T*-tests with the null hypothesis (investments are not more efficient than index portfolio investments) and the alternative hypothesis (investment methods are more efficient).  Table 8 summarizes the results of the tests by *p*-value. The terminologies in Tables 7 and 8 are explained in Table 9.

### 4.5. Factor Affecting the Stock Price Movement

This study performed logistic regression for the entire data to determine the factors affecting the stock price movement. Logistic regression results are shown in Table 10. The regression result in Table 10 shows that the factors HL, LO, variation, vnic, vnipc, insec, and sd7 have a statistically significant impact, of which vnipc has the most substantial impact. This conclusion shows that market portfolio return is the strongest indicator of price change expectations; for every extra percentage rise in market portfolio return, investors anticipate the odds ratio increasing by 0.2. In addition, the model also shows that the moving average indicators (MA) are not statistically significant at 0.1, that is, the MA indicator does not affect stock trading. Volatility indicators HL and LO have regression coefficients of 0.055 and 0.061, respectively. Both are statistically significant, showing that these fluctuations increase the possibility of bullish forecasting for the next trading session. Nevertheless, the vnic indicator has a negative coefficient and is statistically significant, showing that the greater the market volatility, the more it predicts that the price will decrease.

## 5. Discussion

The nonparametric runs test examines the randomness of a sequence of rising/falling states of stock prices. The weak-form efficient market implies that prices rise/fall randomly [66]. This study performs a runs test on two rising/falling ranges of the VN30 and VN-Index portfolios with the null hypothesis that the direction of price movement is random. Runs test results in Table 11 have a *p*-value less than 0.01. This study rejects the null hypothesis for both tests [67]. This result implies that the weak-form efficient-market hypothesis is rejected. This result is also consistent with some previous research [61, 68–70]. Market weakness is not guaranteed to present an opportunity for short-term traders looking for past patterns to rely on when buying/selling to maximize trading profits.

This study implements three trading strategies: the logistic regression model, the SVM model, and holding stocks for the long term. In the first two strategies, the models forecast the increase/decrease of the stock price, resulting in buying and selling correspondingly. Compared to the traditional logistic regression model, the SVM model better predicts price movement direction. On all 30 tickers in Table 6, the SVM model defeated the logistic regression model. Additionally, its accuracy is exceptional, averaging 92.48% and 58.93%. This finding is much like prior studies, which show that SVM produces greater accuracy than the logistic regression model [71–74].

The accuracy of the SVM model in Table 6 is very high, with most of them correct over 90%, except for the two tickers: the VJC ticker and VPB ticker. Moreover, its lowest accuracy is 86.66%, and the highest is 96.94% (for TPB ticker). This result is better than similar studies such as Kim [75], Kara et al. [76], Patel et al. [77], and Duong et al. [78]. One success in the SVM model comes from its model estimation method. Compared to other methods, the “rolling window” is more efficient because the continuous-time series ensures the input parameters' accuracy. The 365-day period is a reasonable choice. If it is longer, the data will become too outdated. If it is shorter, the collected data may not be a good representation of the whole. Specifically, the training data is permanently fixed for the latest 365 observations. Because of the continual updating of the training data set, the initial parameters are adjusted accordingly, increasing the forecasts' accuracy.

In contrast, the sample's representativeness will be a problem if the data set is split into two independent sets. For example, Vijh et al. [55] divided the data set into two sets: the training data set (June 4, 2009–March 4, 2017) and the testing data set (April 4, 2017–May 4, 2019). The parameters calculated by the training data set are too outdated for forecasting; using data from 2017 to forecast for 2019 does not seem to be reasonable. Cao and Tay [21] and Ji et al. [79] divided the data set into three sets: training, validation, and testing data. While rationality is much better when applied historical data, performance will be significantly less than the rolling window.

The superior predictive power of the SVM model has led to excellent trading performance. From Table 7, using the SVM model for trading has achieved an average rate of return of 1.426%/day with the corresponding Sharpe ratio of 0.781, which is much greater than using the logistic regression model. Although the logistic regression method is not as effective as the SVM model, it still produces a great result with an average return rate of 0.348%/day and a Sharpe ratio of 0.146. In contrast, the average rate of return of VN30 and VN-Index is only 0.06% per day and 0.04% per day, respectively. The efficiency test results of all three methods (trading under the SVM model, logistic regression, and long-term holding of individual stocks) in Table 8 suggest that the SVM method is more efficient than investment according to the VN30 and VN-Index with a significance level of 0.001 (the *p*-values are approximately 0). Trading using the logistic regression model is effective when 25 out of 30 stocks achieved statistical significance at 0.1. For long-term holding of individual stocks, the average return rate is 0.052%/day, higher than VN-Index (0.04%/day) but lower than the VN30 index (0.06%/day). Furthermore, the *p*-values are all greater than 0.1, implying that the investing strategy of long-term holding individual stocks cannot outperform the market.

Logistic regression results reveal that indicators such as HL, LO, variation, vnic, vnipc, and sd7 impact stock price movement. Specifically, the increase of HL, LO, vnipc, and sd7 predicts that the price will increase, and VNC ticker increase predicts that the price will decrease. Indicators related to MA and close are not statistically significant and therefore do not have a predictive function of stock price movement.

## 6. Conclusion

Financial markets are efficient when old and new information is quickly reflected in the current price of a security. Therefore, because the current price includes historical information, technical analysis will not guarantee an excess return. Unfortunately, the test results reveal that the HoSE market is inefficient, meaning that technical analysis might generate abnormal returns.

The study's main contributions are identifying weak-form efficient markets and providing trading strategies for short-term investors by applying the machine learning model to optimize profits. Stock price movement forecasting algorithms, particularly the SVM model, have shown the predicting effectiveness, with an average accuracy of up to 92.48% and the peak accuracy of 96.94% (for the ticker TPB). The rolling window approach performed well in predicting the parameters of the machine learning model. The duration of the historical data is critical because the sample's representativeness may be compromised by a period that is too short or too long; hence, 365 days is considered a suitable option. Stock trading in an underperforming market is always a challenge for short-term investors. One trading strategy investors should consider there is the logistic regression model (especially the SVM model) to forecast price movement direction. Because high investment returns often conceal underlying risks, investment results should be adjusted accordingly to increase the confidence level in the investment performance evaluation. This study chooses the Sharpe ratio for risk adjustment and uses the *T*-Test to determine the effectiveness of trading strategies. Due to the high accuracy of the SVM model, the trading strategy using it has earned an exceptional rate of return.

Moreover, as the HoSE stock market is inefficient, short-term investors can rely on past patterns to maximize returns in trading. Short-term investors should consider using the SVM model and logistic regression models when making buying/selling decisions. The decision to choose trading stocks should be based on several indicators such as intraday price movement, price movement between two consecutive trading sessions, moving average, the standard deviation of the stock, and market volatility. It is possible to synthesize the SVM model from those indicators into an indicator for the final forecast. For long-term investors, it is better to invest in a diversified portfolio or a portfolio index rather than holding individual stocks. Medium- to long-term investors should invest in a diversified portfolio or use fundamental analysis to select good stocks for a longer-term plan. Investors with limited knowledge related to pattern analysis can rely on indicators such as intraday price movement, price movement between two straight days, market volatility, and the stock's overall risk in the short term to forecast an increase or decrease in a security's price. Moreover, the return on the market portfolio is the most potent indicator because it reflects an optimistic attitude towards the market. If returns are positive, investors are more optimistic about market growth and thus decide to buy more; as a result, the stock price will increase.

Although this trading method has obtained an unprecedented return rate on short-term trading, the study omitted several factors such as transaction costs, taxes, and liquidity risk. Superior returns also use historical information, which is only valuable for inefficient market conditions. Therefore, more experiments are needed on inefficient markets to increase the reliability of the model. Further research may expand in two directions. First, the model's effectiveness in different markets has to be tested, and other factors such as tax transaction costs has to be considered. Second, the other authors can apply machine learning algorithms such as tree decision, deep learning, and neural networks to increase the model's predictive ability.

## Figures and Tables

**Table 1:** Logistic regression in prior studies.

Authors	Research	Accuracy
Jabeur [46]	Bankruptcy prediction using partial least squares logistic regression	∼70%
Rafatnia et al. [47]	Financial distress prediction across firms	83.3%
Jovanović et al. [48]	Financial indicators as predictors of illiquidity	95.5%
Strzelecka et al. [49]	Application of logistic regression models to assess household financial decisions regarding debt	70.5%

**Table 2:** Tickers and observations in the VN30 basket.

Ticker	Observations	Ticker	Observations	Ticker	Observations
BID	1,840	MWG	1,731	TCB	762
BVH	2,989	NVL	1,112	TCH	1,173
CTG	2,975	PDR	2,573	TPB	789
FPT	3,611	PLX	1,039	VCB	2,987
GAS	2,265	PNJ	3,043	VHM	772
HDB	857	POW	605	VIC	3,424
HPG	3,381	REE	5,050	VJC	1,076
KDH	2,833	SBT	3,319	VNM	3,838
MBB	2,401	SSI	3,600	VPB	957
MSN	2,896	STB	3,720	VRE	898

**Table 3:** Variable description.

Variables	Formula	Description	Source
*close*_*t*_		The closing price at date *t*	Schöneburg [53]
*fore* *di* *r*_*t*_	$fore\;di\;r_{t}=\left\{\begin{array}{c} 1,close_{t}\geq close_{t-1}\\ -1,close_{t}<close_{t-1} \end{array}\right)$	The direction of price movement (foredir = 1 implies that the closing price increases from the previous day)	Ren et al. [54]
*HL*_*t*_	*high*_*t*_ − *low*_*t*_	The fluctuation of the price within a trading day	Vijh et al. [55]
*LO*_*t*_	*low*_*t*−1_ − *open*_*t*_	The difference between the lowest price and the opening price	
*variation*_*t*_	*close*_*t*_ − *close*_*t*−1_	The fluctuation of closing prices between 2 consecutive days	
*ma*7_*t*_	1/7∑_*i*=0_^6^*close*_*t*−*i*_	The average closing price of 7 consecutive trading sessions	
*ma*14_*t*_	1/14∑_*i*=0_^6^*close*_*t*−*i*_	The average closing price of 14 consecutive trading sessions	
*ma*21_*t*_	1/21∑_*i*=0_^6^*close*_*t*−*i*_	The average closing price of 21 consecutive trading sessions	
*s* *d*7_*t*_	$\sqrt{\mathrm{var}\left(close_{t},close_{t-1},\ldots ,close_{t-6}\right)}$	The standard deviation of the closing price of 7 consecutive trading sessions	
*vnic*_*t*_	*vnin* *de* *x*_*t*_ − *vnin* *de* *x*_*t*−1_	Fluctuation of VN-Index between 2 consecutive trading sessions	Qiu and song [56]
*vnipc*_*t*_	*vnin* *de* *x*_*t*_ − *vnin* *de* *x*_*t*−1_/*vnin* *de* *x*_*t*−1_ × 100	The return rate of the VN-Index portfolio	
*insec*_*t*_		Time trend variable (the default origin is January 1, 1970)	

**Table 4:** Descriptive statistics of variables.

Statistics	Close	HL	LO	Variation	ma7	ma14	ma21	sd7
Min	5.3	−161.7	−40	−169	5.39	5.59	5.83	0
Median	37.2	0.8	−0.3	0	37.2	37.25	37.26	0.66
Max	665	44.5	82	30	634.57	623	622.14	93.83
Mean	51.54	1.242	−0.62	0	51.56	51.58	51.59	1.19
SD	43	1.6	1.13	2	43.1	43.2	43.27	2.03

**Table 5:** The statistics of the variables in the market.

Statistics	closevn	vnic	vnipc	closevn30	rvn30	rf
Min	235.5	−73.23	−7.15	230.7	−6.73	0.27
Median	582	0.68	0.1	614.1	0.11	4.86
Max	1,420.3	40.85	4.74	1,557.4	5.16	13.83
Mean	668.7	0.32	0.04	677	0.06	5.77
SD	246.45	8.7	1.28	228.59	1.31	3.49

**Table 6:** A summary on accuracy of tickers in VN30 portfolio.

Ticker	Logistic	SVM	Ticker	Logistic	SVM	Ticker	Logistic	SVM
BID	59.49	92.55	POW	56.85	93.36	MWG	53.84	91.51
BVH	58.4	92.91	REE	62.21	92.53	NVL	58.29	93.72
CTG	61.93	93.53	SBT	62.2	93.6	PDR	59.98	92.76
FPT	58.67	90.64	SSI	58.9	91.75	PLX	56.3	91.41
GAS	57.55	92.9	STB	63.32	92.19	PNJ	57.86	91.64
HDB	57.61	93.71	TCB	55.53	95.73	VIC	59.87	91.57
HPG	58.93	91.22	TCH	54.88	91.97	VJC	58.57	86.66
KDH	61.89	93.36	TPB	58.82	96.94	VNM	61.83	91.34
MBB	62.15	93.67	VCB	58.6	93.25	VPB	57.5	88.03
MSN	62.48	93.05	VHM	57.84	94.85	VRE	55.62	91.95
Average accuracy		SVM				Logistic		
		92.48				58.93		

**Table 7:** Stock trading results.

Ticker	SVM	Logistic	Return	Adj SVM	Adj logistic	Adj return
BID	1.448	0.177	0.067	0.780	0.072	0.025
BVH	1.450	0.280	0.038	0.800	0.116	0.010
CTG	1.432	0.380	0.046	0.788	0.162	0.014
FPT	1.353	0.331	0.054	0.726	0.135	0.015
GAS	1.421	0.257	0.068	0.775	0.108	0.026
HDB	1.469	0.465	0.059	0.839	0.207	0.025
HPG	1.333	0.316	0.048	0.705	0.132	0.014
KDH	1.427	0.427	0.039	0.781	0.183	0.012
MBB	1.462	0.398	0.067	0.811	0.170	0.025
MSN	1.434	0.449	0.038	0.793	0.194	0.011
MWG	1.359	0.211	0.094	0.716	0.088	0.037
NVL	1.411	0.284	0.083	0.808	0.125	0.035
PDR	1.494	0.396	0.062	0.812	0.166	0.022
PLX	1.240	0.226	0.065	0.702	0.104	0.028
PNJ	1.375	0.329	0.038	0.720	0.136	0.010
POW	1.344	0.317	0.034	0.888	0.158	0.017
REE	1.552	0.467	0.053	0.730	0.134	0.013
SBT	1.427	0.411	0.049	0.778	0.174	0.015
SSI	1.372	0.309	0.053	0.748	0.127	0.015
STB	1.402	0.425	0.052	0.721	0.160	0.014
TCB	1.603	0.271	0.006	0.864	0.111	0.001
TCH	1.475	0.242	0.056	0.784	0.100	0.021
TPB	1.643	0.419	0.027	0.936	0.176	0.010
VCB	1.452	0.325	0.041	0.803	0.136	0.012
VHM	1.590	0.477	0.035	0.859	0.198	0.014
VIC	1.396	0.369	0.049	0.758	0.156	0.015
VJC	1.268	0.367	0.047	0.690	0.165	0.019
VNM	1.437	0.460	0.063	0.743	0.176	0.014
VPB	1.316	0.305	0.045	0.755	0.139	0.019
VRE	1.397	0.355	0.071	0.802	0.160	0.030
Average	1.426	0.348	0.052	0.781	0.146	0.018

**Table 8:** The *p*-value for testing the effectiveness of trading methods.

Ticker	p_LR_vn30	p_LR_vni	p_SVM_vn30	p_SVM_vni	p_return
BID	0.291	0.178	0.001	0.001	0.641
BVH	0.001	0.001	0.001	0.001	0.549
CTG	0.001	0.001	0.001	0.001	0.499
FPT	0.001	0.001	0.001	0.001	0.543
GAS	0.020	0.010	0.001	0.001	0.578
HDB	0.020	0.004	0.001	0.001	0.574
HPG	0.001	0.001	0.001	0.001	0.464
KDH	0.001	0.001	0.001	0.001	0.628
MBB	0.001	0.001	0.001	0.001	0.531
MSN	0.001	0.001	0.001	0.001	0.643
MWG	0.224	0.131	0.001	0.001	0.581
NVL	0.069	0.027	0.001	0.001	0.428
PDR	0.001	0.001	0.001	0.001	0.666
PLX	0.211	0.103	0.001	0.001	0.550
PNJ	0.001	0.001	0.001	0.001	0.599
POW	0.615	0.387	0.001	0.001	0.897
REE	0.001	0.001	0.001	0.001	0.562
SBT	0.001	0.001	0.001	0.001	0.346
SSI	0.001	0.001	0.001	0.001	0.542
STB	0.001	0.001	0.001	0.001	0.549
TCB	0.377	0.178	0.001	0.001	0.730
TCH	0.092	0.038	0.001	0.001	0.419
TPB	0.076	0.021	0.001	0.001	0.651
VCB	0.001	0.001	0.001	0.001	0.533
VHM	0.055	0.014	0.001	0.001	0.667
VIC	0.001	0.001	0.001	0.001	0.540
VJC	0.012	0.003	0.001	0.001	0.510
VNM	0.001	0.001	0.001	0.001	0.549
VPB	0.096	0.030	0.001	0.001	0.572
VRE	0.088	0.024	0.001	0.001	0.552

**Table 9:** Glossary of variables.

Variable	Definition
SVM	The average rate of return using the SVM model
Logistic	The average rate of return using the logistic regression model
Return	The average rate of return on investment for holding securities
Adj SVM, adj logistic, and adj return	Risk-adjusted rate of return
p_LR_vn30, p_LR_vni, p_SVM_vn30, and p_SVM_vni	The *p*-value for testing if LR and SVM methods are more effective than investing by VN30 index and VN-Index
p_return	The *p*-value for testing the efficiency of holding a single stock compared to holding the VN-Index

**Table 10:** Logistic regression result.

Deviance residuals				
Min	1Q	Median	3Q	Max
–2.2376	−1.0705	−0.9763	1.2765	1.9416
	Estimate	Std. error	*z* value	Pr(>|z|)
(Intercept)	−1.54*e + *00	8.77*e − *02	−17.589	<2*e − *16^*∗∗∗*^
close	3.57*e − *03	4.41*e − *03	0.81	0.4179
HL	5.53*e − *02	9.29*e − *03	5.951	2.67*e − *09^*∗∗∗*^
LO	6.07*e − *02	1.11*e − *02	5.47	4.50*e − *08^*∗∗∗*^
variation	1.00*e − *02	6.00*e − *03	1.67	0.0949
vnicbb	−2.51*e − *02	2.18*e − *03	−11.543	<2*e − *16^*∗∗∗*^
vnipc	2.00*e − *01	1.63*e − *02	12.295	<2*e − *16^*∗∗∗*^
insec	7.45*e − *05	5.18*e − *06	14.389	<2*e − *16^*∗∗∗*^
ma7	4.88*e − *03	7.81*e − *03	0.625	0.5321
ma14	−1.34*e − *03	9.15*e − *03	−0.146	0.8838
ma21	−8.35*e − *03	5.35*e − *03	−1.562	0.1184
sd7	3.98*e − *02	5.54*e − *03	7.184	6.76*e − *13^*∗∗∗*^

*Note.* Significance codes: 0 “*∗∗∗*“ 0.001 “*∗∗* 0.01 “*∗*” 0.05 “.” 0.1 “ “ 1.

**Table 11:** Runs tests results.

VN30 index	VN-Index
Approximate runs rest data: VN30	Approximate runs rest data: VN-Index
Runs = 1,482; *p*-value = 0.006332; alternative hypothesis: two-sided	Runs = 1,466; *p*-value = 0.001233; alternative hypothesis: two-sided

## Data Availability

The data are available on request.
